# Gray-Scale vs. Color-Doppler Ultrasound in Pediatric Fatty Liver: A Diagnostic Disparity

**DOI:** 10.34172/mejdd.3321

**Published:** 2026-01-31

**Authors:** Amirreza Jahanshahi, Maryam Shoaran, Sina Raeisi, Mahla Miri, Khatereh Rezazadeh

**Affiliations:** ^1^Department of Radiology, Imam Reza Hospital, Faculty of Medicine, Tabriz University of Medical Sciences, Tabriz, Iran; ^2^Pediatric Health Research Center, Zahra Mardani Azari Children Hospital, Tabriz University of Medical Sciences, Tabriz, Iran

**Keywords:** Fatty liver, Gray-scale ultrasound, Color-doppler, Pediatric obesity, Liver enzymes, Non-invasive diagnosis

## Abstract

**Introduction:**

Fatty liver disease is a growing cause of chronic liver disorders in children, largely attributed to the increasing prevalence of obesity. Accurate, non-invasive diagnosis is important to prevent progression to fibrosis or cirrhosis. While liver biopsy is the gold standard, ultrasonography remains the most widely used diagnostic tool. This study compared the diagnostic efficacy of Gray-scale and color-Doppler ultrasound, alongside biochemical markers, in overweight and obese children.

**Methods:**

In this cross-sectional study, 67 children aged 2-15 years with body mass index (BMI) above the 85th percentile underwent both Gray-scale and color-Doppler ultrasound examinations. Serum levels of cholesterol, triglyceride, alanine aminotransferase (ALT), aspartate aminotransferase (AST), and other liver-related biochemical parameters were also assessed.

**Results:**

Gray-scale ultrasound detected fatty liver in 41.79% of the children, with 29.85% having grade 1 and 11.94% grade 2 steatosis. Color-Doppler ultrasound revealed no diagnostic abnormalities. Serum ALT, AST, cholesterol, and triglyceride levels were significantly elevated in children with sonographic evidence of fatty liver compared with those with normal findings (*P*<0.01), though no differences were found between grades 1 and 2.

**Conclusion:**

Gray-scale ultrasound is effective for diagnosing pediatric fatty liver, whereas color-Doppler imaging adds no diagnostic benefit. Biochemical markers, while useful, lack sensitivity in differentiating disease severity.

## Introduction

 Fatty liver is one of the chronic liver disorders that occurs if fat accumulates in more than 5% of the liver weight in hepatocytes.^1,2^ The number of patients with this disease is increasing due to various reasons, such as lifestyle changes, decreased physical activity, and poor eating habits.^3,4,5^ Most people with fatty liver disease suffer from obesity, diabetes mellitus, hyperlipidemia, and hypertension.^6^

 Non-alcoholic fatty liver disease (NAFLD) is increasingly recognized in children and adolescents, paralleling the global rise in pediatric obesity. Its prevalence in children ranges from 5–15% in community-based cohorts to more than 40% in obese populations.^7,8,9^ Accurate and timely diagnosis is essential, since untreated pediatric fatty liver may progress to steatohepatitis, fibrosis, and, rarely, cirrhosis or hepatocellular carcinoma.^10,11^

 Although liver biopsy remains the diagnostic gold standard, it is invasive and unsuitable for routine use in children.^12,13^ Ultrasonography, particularly Gray-Scale imaging, is therefore widely used as a first-line, non-invasive tool. Gray-scale ultrasound provides qualitative information about hepatic echogenicity, which correlates with fat infiltration.^14,15,16^

 Color-Doppler ultrasound, though not designed to detect fat infiltration, can evaluate hepatic blood flow. Previous studies have examined Doppler indices in pediatric and adolescent patients with NAFLD to investigate possible hemodynamic alterations. For instance, Riahinezhad and colleagues assessed the Doppler perfusion index in adolescents with NAFLD and found no difference compared with healthy subjects, although some portal vein flow parameters were significantly lower in affected patients.^7^ These findings highlight ongoing interest in the potential vascular correlates of fatty liver, even if diagnostic utility remains limited.

 The present study aimed to compare Gray-scale and color-Doppler ultrasonography in the diagnosis of fatty liver in overweight and obese children, and to evaluate their relationship with biochemical markers. To our knowledge, few studies have assessed these modalities together in a pediatric population, which underscores the exploratory nature and novelty of our research.

## Materials and Methods

###  Patients 

 In the present cross-sectional study, among children aged 2-15 years referred to Tabriz Children Hospital during 2019, 67 available children with obesity criteria [body mass index (BMI) greater than 95th percentile] or overweight (BMI between 85th and 95th percentile) were included in the study after obtaining written consent from their parents. These children with the possibility of fatty liver underwent Gray-scale and color-Doppler sonography.

###  Imaging Analysis

 Based on the Gray-scale ultrasound image, Fatty liver was graded 1-3 (0 = normal). Grade 1, as a mild type, was characterized by a sparse, diffuse increase in liver echoes, with normal visualization of intrahepatic vessels and the diaphragm. Grade 2, as a moderate type, was considered if a moderate and diffuse increase in the echoes with slightly impaired visualization of the diaphragm and intrahepatic vessels was detected. Grade 3, as a severe type, was represented by an intense increase in echoes with poor or non-visualization of the diaphragm, intrahepatic vessels, and posterior right lobe of the liver.^17^

###  Laboratory Examinations

 Blood samples were taken from the patients and centrifuged at 3000 g for 10 minutes. The sera were separated and stored at -70 °C in a freezer until analysis. After sample collection, serum levels of biochemical markers, including cholesterol, triglyceride, total and direct bilirubin, alkaline phosphatase (ALP), aspartate aminotransferase (AST), and alanine aminotransferase (ALT), were evaluated by a biochemical autoanalyzer (Selectra 2, Vitalab, Netherlands).

###  Statistical analyses

 The data were statistically analyzed by SPSS software (version 16.0; SPSS, Inc., Chicago, IL). Initially, the variables were statistically checked for normality by a one-sample Kolmogorov–Smirnov test. All variables except total and direct bilirubin had normal distributions and therefore were shown as mean ± standard deviation and compared by one-way ANOVA followed by Tukey’s post-hoc tests between sonographically normal (grade 0), grade 1, and grade 2 fatty liver cases. Non-parametric variables, including sex, Gray-scale, and color-Doppler findings, were shown as percentages. Total and direct bilirubin were shown as median with interquartile range and compared by the Mann-Whitney U-test. A P value of less than 0.05 was considered statistically significant.

## Results

 In the present study, 67 children with obesity or overweight criteria were included.

 The study participants consisted of 33 (49.25%) boys and 34 (50.75%) girls, with a mean age of 7.94 ± 3.49. The calculated BMI of the children was 21.29 ± 3.35 (kg/m^2^). Demographic characteristics and sonography results of the studied children are depicted in Table 1.

**Table 1:** Demographic characteristics and sonography results of the children

**Parameters**	
Age (year)	7.94 ± 3.49
Sex (F/M)	34/33
BMI (Kg/m^2^)	21.29 ± 3.35
Gray-scale US	
Grade 0 (Normal) (n/%)	39 (58.21)
Grade 1 Fatty liver (n/%)	20 (29.85)
Grade 2 Fatty liver (n/%)	8 (11.94)
Grade 3 Fatty liver (n/%)	0 (0)
Color-Doppler	
Normal (n/%)	67 (100)
Liver abnormality (n/%)	0 (0)

BMI, body mass index; F, female; M, male; US, ultrasound

 According to Gray-scale imaging, of 67 studied children, fatty liver was diagnosed in 28 (41.79%) patients, in which 20 cases (29.85%) had grade 1, and 8 cases (11.94%) suffered from grade 2 fatty liver. No children with grade 3 fatty liver were found among the studied children, and the remaining 39 individuals (58.21%) had sonographically normal livers. No abnormality was found in any of the subjects by color-Doppler imaging. Therefore, the agreement between color-Doppler and Gray-scale ultrasound findings could not be statistically evaluated. The comparison of serum biochemical parameters between the groups is shown in Table 2. As shown in the table, serum levels of ALT and AST in grade 1 (67.65 ± 29.59, 76.60 ± 29.93; U/L; respectively) and grade 2 (83.87 ± 17.88, 96.25 ± 19.23; U/L; respectively) fatty liver patients were significantly (*P* < 0.01) higher than those (30.36 ± 12.51, 31.59 ± 12.56; U/L; respectively) in sonographically normal liver individuals.

**Table 2:** The comparison of the parameters between the Grade 0 (Normal), Grade 1, and Grade 2 groups. ALT, alanine aminotransferase; AST, aspartate aminotransferase; ALP, alkaline phosphatase; BMI, body mass index; Ch, Cholesterol; D. Bil, direct Bilirubin; T. Bil, total bilirubin; TG, triglyceride.

**Parameters**	**Grade 0 **	**Grade 1**	**Grade 2**	***P*** ** value**
Age (year)	8.10 ± 3.49	7.25 ± 3.65	8.87 ± 3.18	0.653^a^, 0.838^b^, 0.513^c^
BMI (kg/m2)	21.46 ± 3.36	20.40 ± 3.43	22.72 ± 2.73	0.480^a^, 0.593^b^, 0.0.224^c^
ALT (U/L)	30.36 ± 12.51	67.65 ± 29.59	83.87 ± 17.88	< 0.01^a^*, < 0.01^b^*, 0.128^c^
AST (U/L)	31.59 ± 12.56	76.60 ± 29.93	96.25 ± 19.23	< 0.01^a^*, < 0.01^b^*, 0.056^c^
ALP (U/L)	675.38 ± 213.47	803.10 ± 144.88	838.62 ± 248.69	0.060^a^, 0.098^b^, 0.906^c^
T. Bil (mg/dL)	1 (0.7 – 1.1)	1 (0.72 – 1)	1 (0.57 – 1.17)	0.935^a^, 0.751^b^, 0.695^c^
D. Bil (mg/dL)	0.2 (0.2 – 0.2)	0.2 (0.2 – 0.2)	0.2 (0.12 – 0.27)	0.879^a^, 0.895^b^, 0.839^c^
Ch (mg/dL)	106.92 ± 41.17	157.30 ± 38.50	181.25 ± 33.67	< 0.01^a^*, < 0.01^b^*, 0.324^c^
TG (mg/dL)	95.85 ± 41.01	169.00 ± 38.65	188.75 ± 45.49	< 0.01^a^*, < 0.01^b^*, 0.484^c^

^a^The comparison between Grade 1 and Grade 0; ^b^The comparison between Grade 2 and Grade 0; ^c^The comparison between Grade 1 and Grade 2 groups. *Statistically significant (*P* < 0.05).

 Serum levels of cholesterol and triglyceride in grade 1 (157.30 ± 38.50, 169.00 ± 38.65; mg/dL; respectively) and grade 2 (181.25 ± 33.67, 188.75 ± 45.49; mg/dL; respectively) fatty liver patients were significantly (*P* < 0.01) higher than those (106.92 ± 41.17, 95.85 ± 41.01; mg/dL; respectively) in sonographically normal liver individuals. The differences in these parameters were not significant (*P* > 0.05) between the grade 1 and grade 2 groups. No other significant differences were found in the evaluated biochemical markers between the groups (*P* > 0.05).

## Discussion

 In this cross-sectional pediatric cohort, Gray-scale ultrasound detected fatty liver in 41.79% of children, while color-Doppler revealed no abnormalities. Children with sonographically diagnosed fatty liver had significantly higher ALT, AST, cholesterol, and triglyceride levels compared with those with normal liver ultrasound, although these markers did not distinguish between grade 1 and grade 2 disease.

 Pediatric fatty liver disease is the most prominent cause of chronic liver disease in children. ^18^ Although the gold standard for fatty liver diagnosis is liver biopsy, it may not be suitable for all patients due to its high cost and invasiveness.^19^ Thus, ultrasonography remains the most widely used first-line modality.^20^ Prior studies, such as those by Lewis et al. and Hamaguchi et al., have demonstrated ultrasound’s high diagnostic accuracy compared with biopsy and CT, supporting its clinical utility.^5,21^ These findings confirm that Gray-scale ultrasound remains effective for non-invasive diagnosis in children.

 In the present study, based on Gray-scale ultrasound results, of 67 studied children, fatty liver was diagnosed in 28 (41.79%) patients, of which 20 cases (29.85%) had grade 1, and 8 cases (11.94%) suffered from grade 2 fatty liver. The remaining 39 individuals (58.21%) had sonographically normal liver. However, no abnormality was found in any of the patients on color Doppler imaging. Therefore, the agreement between color-Doppler and Gray-scale ultrasound findings could not be statistically evaluated. The inclusion of color-Doppler in our study was exploratory. Although Doppler ultrasound is primarily a vascular tool, prior research suggested that hemodynamic changes may accompany NAFLD. Riahinezhad and others evaluated Doppler perfusion index in adolescents and found no diagnostic difference compared with controls, though portal vein flow parameters were reduced in affected patients.^7^ Other Doppler-based studies in adults and children have reported altered hepatic or portal vein flow in NAFLD, but their results have been inconsistent. Our findings align with these observations and indicate that color-Doppler does not provide additional diagnostic value in routine pediatric fatty liver assessment.

 Due to the association of fatty liver with hyperlipidemia and also the possibility of liver damage, the evaluation of some related serum biomarkers such as lipid profile and liver enzymes can also be helpful in the prediction and diagnosis of fatty liver.^22,23^ In a study by Angelico and colleagues ^24^, hypertriglyceridemia was the main feature of patients with fatty liver. Serum levels of ALT and AST were also significantly higher than in healthy individuals. Ni and others ^25^ found that 41.2% and 14.7% of patients with fatty liver had hypertriglyceridemia and hypercholesterolemia, respectively. Also, serum ALT and AST in the patients were significantly higher than in the healthy controls. In the present study, serum levels of cholesterol, triglyceride, ALT, and AST in grade 1 and grade 2 fatty liver patients were significantly (*P* < 0.01) higher than those in the individuals with sonographically normal liver. However, these parameters were not significantly different between grade 1 and grade 2 groups. The enzymes ALT and AST are commonly considered as “liver tests” which increase during liver damage, however, they are not liver-specific, being found in liver, muscle, red blood cells, and other tissues.^26^ The results of the present study also indicated that although ALT and AST elevation could be caused by lipid accumulation and subsequent liver damage, they might be not sensitive enough to differentiate grade 1 and grade 2 fatty liver.

 This study contributes to the limited pediatric NAFLD literature by combining imaging and biochemical approaches. Nevertheless, several limitations should be acknowledged: the absence of liver biopsy confirmation, which is the diagnostic gold standard but ethically challenging in children; the relatively small sample size; and the cross-sectional design, which limits conclusions about disease progression. Future studies with larger cohorts should incorporate histology or non-invasive alternatives such as elastography, MRI-proton density fat fraction (MRI-PDFF), or controlled attenuation parameter (CAP) to validate and extend these findings.

## Conclusion

 Gray-scale ultrasound remains a reliable non-invasive tool for diagnosing pediatric fatty liver, whereas color-Doppler adds no diagnostic benefit. Our results, consistent with prior Doppler studies, suggest that vascular assessment should not be routinely employed for diagnosis. Instead, future diagnostic strategies in children with suspected NAFLD should focus on combining gray-scale ultrasound with advanced imaging and biochemical markers to improve accuracy and staging.

## References

[1] Anderson EL, Howe LD, Jones HE, Higgins JP, Lawlor DA, Fraser A (2015). The Prevalence of Non-Alcoholic Fatty Liver Disease in Children and Adolescents: A Systematic Review and Meta-Analysis. PLoS One.

[2] Schwimmer JB, Deutsch R, Kahen T, Lavine JE, Stanley C, Behling C (2006). Prevalence of Fatty Liver in Children and Adolescents. Pediatrics.

[3] Rinella ME (2015). Nonalcoholic fatty liver disease: a systematic review. Jama.

[4] Vajro P, Lenta S, Socha P, Dhawan A, McKiernan P, Baumann U (2012). Diagnosis of nonalcoholic fatty liver disease in children and adolescents: position paper of the ESPGHAN Hepatology Committee. J Pediatr Gastroenterol Nutr.

[5] Hamaguchi M, Kojima T, Itoh Y, Harano Y, Fujii K, Nakajima T (2007). The severity of ultrasonographic findings in nonalcoholic fatty liver disease reflects the metabolic syndrome and visceral fat accumulation. Am J Gastroenterol.

[6] Hernaez R, Lazo M, Bonekamp S, Kamel I, Brancati FL, Guallar E (2011). Diagnostic accuracy and reliability of ultrasonography for the detection of fatty liver: a meta-analysis. Hepatology.

[7] Riahinezhad M, Saneian H, Farghadani M, Parsai Arshad S (2021). Doppler perfusion index in adolescents with non-alcoholic fatty liver disease. Immunopathol Persa.

[8] Pasarín M, Abraldes JG, Liguori E, Kok B, La Mura V (2017). Intrahepatic vascular changes in non-alcoholic fatty liver disease: Potential role of insulin-resistance and endothelial dysfunction. World J Gastroenterol.

[9] Nasiri-Ansari N, Androutsakos T, Flessa CM, Kyrou I, Siasos G, Randeva HS (2022). Endothelial Cell Dysfunction and Nonalcoholic Fatty Liver Disease (NAFLD): A Concise Review. Cells.

[10] Szczepaniak LS, Nurenberg P, Leonard D, Browning JD, Reingold JS, Grundy S (2005). Magnetic resonance spectroscopy to measure hepatic triglyceride content: prevalence of hepatic steatosis in the general population. Am J Physiol Endocrinol Metab.

[11] Kleiner DE, Brunt EM (2012). Nonalcoholic fatty liver disease: pathologic patterns and biopsy evaluation in clinical research. Semin Liver Dis.

[12] Angelico F, Del Ben M, Conti R, Francioso S, Feole K, Fiorello S (2005). Insulin resistance, the metabolic syndrome, and nonalcoholic fatty liver disease. J Clin Endocrinol Metab.

[13] Kakkos SK, Yarmenitis SD, Tsamandas AC, Gogos CA, Kalfarentzos F (2000). Fatty liver in obesity: relation to Doppler perfusion index measurement of the liver. Scand J Gastroenterol.

[14] Balasubramanian P, Boopathy V, Govindasamy E, Venkatesh BP (2016). Assessment of Portal Venous and Hepatic Artery Haemodynamic Variation in Non-Alcoholic Fatty Liver Disease (NAFLD) Patients. J Clin Diagn Res.

[15] Uzun H, Yazici B, Erdogmus B, Kocabay K, Buyukkaya R, Buyukkaya A (2009). Doppler waveforms of the hepatic veins in children with diffuse fatty infiltration of the liver. Eur J Radiol.

[16] Balci A, Karazincir S, Sumbas H, Oter Y, Egilmez E, Inandi T (2008). Effects of diffuse fatty infiltration of the liver on portal vein flow hemodynamics. J Clin Ultrasound.

[17] Mahale AR, Prabhu SD, Nachiappan M, Fernandes M, Ullal S (2018). Clinical relevance of reporting fatty liver on ultrasound in asymptomatic patients during routine health checkups. J Int Med Res.

[18] Whitsett M, VanWagner LB (2015). Physical activity as a treatment of non-alcoholic fatty liver disease: A systematic review. World J Hepatol.

[19] Alkassabany YM, Farghaly AG, El-Ghitany EM (2014). Prevalence, risk factors, and predictors of nonalcoholic fatty liver disease among schoolchildren: a hospital-based study in Alexandria, Egypt. Arab J Gastroenterol.

[20] Sattar N, Forrest E, Preiss D (2014). Non-alcoholic fatty liver disease. BMJ: British Medical Journal.

[21] Lewis JR, Mohanty SR (2010). Nonalcoholic fatty liver disease: a review and update. Dig Dis Sci.

[22] Neuman MG, Cohen LB, Nanau RM (2014). Biomarkers in nonalcoholic fatty liver disease. Can J Gastroenterol Hepatol.

[23] Bessissow T, Le NH, Rollet K, Afif W, Bitton A, Sebastiani G (2016). Incidence and Predictors of Nonalcoholic Fatty Liver Disease by Serum Biomarkers in Patients with Inflammatory Bowel Disease. Inflamm Bowel Dis.

[24] Angelico F, Del Ben M, Conti R, Francioso S, Feole K, Maccioni D (2003). Non-alcoholic fatty liver syndrome: a hepatic consequence of common metabolic diseases. J Gastroenterol Hepatol.

[25] Ni Y, Liu H, Hu D, Zhe W, Li M (2001). Clinicopathological analysis of non‐alcoholic steatohepatitis. Chinese Journal of Digestive Diseases.

[26] Webster GF, Webster TG, Grimes LR (2017). Laboratory tests in patients treated with isotretinoin: occurrence of liver and muscle abnormalities and failure of AST and ALT to predict liver abnormality. Dermatol Online J.

